# Protection and Controlled Gastrointestinal Release of Bromelain by Encapsulating in Pectin–Resistant Starch Based Hydrogel Beads

**DOI:** 10.3389/fbioe.2021.757176

**Published:** 2021-10-29

**Authors:** Thatchajaree Mala, Anil Kumar Anal

**Affiliations:** Food Engineering and Bioprocess Technology Program, Department of Food, Agriculture and Bioresources, School of Environment, Resources, and Development, Asian Institute of Technology, Pathum Thani, Thailand

**Keywords:** encapsulation, hydrogel bead, bromelain, controlled release, pectin, hi-maize starch

## Abstract

Hybrid pectin and resistant starch–based hydrogel beads loaded with bromelain using the extrusion gelation method were prepared and evaluated to enhance the activity of bromelain during gastrointestinal passage and thermal processing. The solutions of pectin–resistant starch with bromelain were dropped into the gelation bath containing calcium chloride (0.2 M) solution to develop various types of hydrogel beads. The physicochemical characteristics of the synthesized hydrogel beads were evaluated. The ratio (4.5:1.5 w/w) of pectin and resistant starch concentration significantly (*p* < 0.05) enhanced the encapsulation efficiency (80.53%). The presence of resistant starch resulted in increased entrapment of bromelain, improved swelling properties with sustained release behavior, and improved gastric stability than pectin hydrogels alone. The swelling of hydrogel beads was higher at pH 7.4 than pH 1.2. Optimized batch of hybrid pectin/resistant starch exhibited a spherical shape. Optical and scanning electron microscopy showed a more packed and spherical shape from the pectin/resistant starch hydrogel bead network. Fourier transformation infrared spectroscopy was also used to confirm the presence of bromelain in the hydrogel beads. The encapsulated bromelain in the pectin/hi-maize starch beads produced at a pectin/hi-maize ratio of 4.5:1.5 (percent w/w; formulation P4) obtained the highest relative bromelain activity in all heat treatments including at 95°C, whereas the highest activity of free bromelain was found only at 30°C. Bromelain encapsulated in hydrogels released at a faster rate at simulated intestinal fluid (SIF, pH 7.4) than at simulated gastrointestinal fluid (SGF, pH 1.2).

## Introduction

There has been a growing interest in recent years on bioactive compounds from agro-industrial waste for their health benefits (Ghosh et al., 2019; Náthia-Neves and Alonso, 2021). The bioactive proteins/peptides have recently been developed as effective nutraceuticals and in functional foods, such as bioactive peptides obtained from *Pterophylla beltrani* (Bolivar & Bolivar), peptides from leatherjacket (*Meuchenia* sp.), protein hydrolysates, and bioactive proteolytic enzymes derived from pineapple (Salampessy et al., 2017; Montiel-Aguilar et al., 2020; Chakraborty et al., 2021). Bromelain, a protease enzyme, is abundant in the pineapple fruit pulp and its byproducts (Ketnawa et al., 2012; Mala et al., 2021a). In the food industry, it is used for meat tenderization, grain protein solubilization during the baking process, beer clarification, and as a supplement (Tochi et al., 2008). Bromelain has also been found effective as a therapeutic agent, including as a suppressor of tumor growth and as an antibacterial, anti-inflammatory, anticoagulant, antiemetic, and antidiarrheal agent (Siow and Lee, 2012; Secor et al., 2013). In cosmetics industries, bromelain is used as an active ingredient in various body care products to provide gentle peeling effects (Aehle, 2007). Bromelain is also used for skin pre-tanning, softening and bating, improving the dyeing properties of protein fibers, and decomposing or partially solubilizing protein fibers from silk and wool (Koh et al., 2006).

However, bromelain is sensitive to extreme conditions such as high temperature, gastric proteases in stomach juice, high acidity, and organic solvents, and thus, reduces its functionalities and bioavailability (Xue et al., 2010; Mala et al., 2021a). Its instability under such stress conditions reduce its enzymatic activity, decrease its health benefits, and limit its pharmacological applications. Susceptibility of protein/peptides to enzymatic degradation, acidic hydrolysis, extending their short half-life, and minimizing passage time in the gastrointestinal tract continue to be major challenges in developing these protein/peptides with optimum bioavailability (Dumont et al., 2019; Kuhlmann et al., 2021). As a result, an optimal and effective oral distribution system for such bioactive ingredients are required for controlled gastrointestinal passage and targeted delivery with improved bioavailability (Anal and Stevens, 2005; Giroux et al., 2016; Ozel et al., 2020).

The encapsulation technique has proven to be a reliable and effective approach for the entrapment of bioactive ingredients into carrier matrices (Bernela et al., 2016; Lan et al., 2021; Marcillo-Parra et al., 2021). Encapsulation has the potential to enhance the protection against severe gastrointestinal conditions (Zhang et al., 2017; Gao et al., 2021). Various carbohydrates such as pectin, alginate, chitosan, starch, and k-carrageenan have shown effective encapsulation matrices for bioactive molecules (Bernela et al., 2016; Zhou et al., 2018; Sun et al., 2020).

Pectin, a high-value functional component in foods, has received significant recognition for probiotic encapsulation in food products being natural, non-toxic, low cost, and biocompatible (Dafe et al., 2017). Pectin is an acidic heteropolysaccharide consisting mainly of D-galacturonic acids linked by 1,4-glycosidic bonds and neutral monosaccharides such as D-arabinose, D-galactose, and L-rhamnose. Pectin is resistant to intestinal or gastric enzymes such as amylase and protease present in the upper gastrointestinal tract. However, it is easily digestible by pectinases produced by the colonic microflora (Wang et al., 2007). Pectin rapidly swells in an aqueous condition due to the presence of several hydroxyl groups. Some natural hydrophobic polymers, including chitosan and hydroxypropyl methylcellulose, have been used to modify pectin to retard its swelling and thus to reduce the release of entrapped bioactive compounds in the stomach (Rampino et al., 2016; Xiang et al., 2020). Similarly, starch, being an inexpensive, abundantly available, and edible polysaccharide has a long history as an excipient in drug compositions (Shrestha et al., 2018; Zhu et al., 2019). Furthermore, the incorporation of starch into the encapsulation matrix results in increased chemical and mechanical stability, improved encapsulation efficiency, and sustained release of different active compounds.

The encapsulation of bromelain in a hydrogel beads comprised of pectin and resistant starch (Hi-maize) as wall materials to protect it from higher processing temperature and its controlled passage and release in the simulated gastrointestinal tract (GIT). This study thus aims to evaluate the effects of resistant starch (Hi-maize) on the activity and controlled delivery of bromelain microencapsulated with the pectin in the simulated digestive system and higher processing temperatures. Hi-maize starch, a modified resistant starch, is considered along with pectin which remains as macromolecular aggregates in the upper portions of GIT. The application of these two biomolecules forms a densely packed matrix (superior protection and tightly packed hydrogel beads) in which the active compounds such as bromelain can be embedded for protection against GIT and thermal conditions.

## Materials and Methods

### Materials

Low methoxyl pectin (LMP, degree of esterification 26%) from citrus peel was obtained from Ingredient Flavours Co., Ltd., Thailand. Hi-maize starch was procured from National Starch and Chemical Co., Ltd., Bangkok, Thailand. Bromelain from pineapple was purchased from Sisco Research Laboratories Pvt. Ltd., India. Pancreatin enzyme was purchased from Sigma-Aldrich, United States, and Pepsin from Acros, Denmark. All other chemicals used in the study were of analytical grade.

### Preparation of Pectin/Hi-Maize Starch Hydrogel Beads Containing Bromelain

Pectin/hi-maize starch hydrogel beads were prepared by gelation method adopting the protocol described by Praepanitchai et al. (2019), with slight modifications (Table 1). Pectin solutions of different concentrations (6, 5.5, 5, 4.5, and 4% w/v) were prepared in distilled water at room temperature (25°C). Hi-maize starch solutions of different concentrations (0, 0.5, 1, 1.5, and 2% w/v) were prepared separately by dissolving them in distilled water at room temperature (25°C). Various ratios of homogenous aqueous solutions of pectin and hi-maize starch (as shown in Table 1) were mixed together at room temperature (25°C). For the preparation of bromelain-loaded pectin/hi-maize starch, the solution of bromelain (100 mg/ml) in a phosphate buffer solution (pH 7) was added in dropwise to the pectin/hi-maize starch solution under constant stirring (200 rpm) on the digital hotplate with a stirrer (UC152D, Stuart, United Kingdom) at room temperature (25°C).

**TABLE 1:** Properties of various pectin/hi-maize starch hydrogel beads.

Formulation	Pectin: Hi-maize starch ratio (% w/w)	Mean size (µm)	Encapsulation efficiencies (%)	DT (h)
P1	6 : 0	4115 ± 99^a^	67.66 ± 2.06^c^	1
P2	5.5 : 0.5	4091 ± 112^a^	71.29 ± 0.99^bc^	1–2
P3	5 : 1	3948 ± 99^b^	73.60 ± 1.51^b^	2–3
P4	4.5 : 1.5	3930 ± 58^bc^	80.53 ± 1.51^a^	>3
P5	4 : 2	3873 ± 95^c^	71.62 ± 1.51^bc^	2–3

DT; disintegration time (h) in simulated intestinal fluid (SIF) with pancreatin, prior incubated in simulated gastric fluid (SGF) for 2 h. Data were expressed as a mean ± standard deviation. The data in the same column with different superscript letters (a, b, c) indicates significant difference at *p* < 0.05.

**FIGURE 1:**
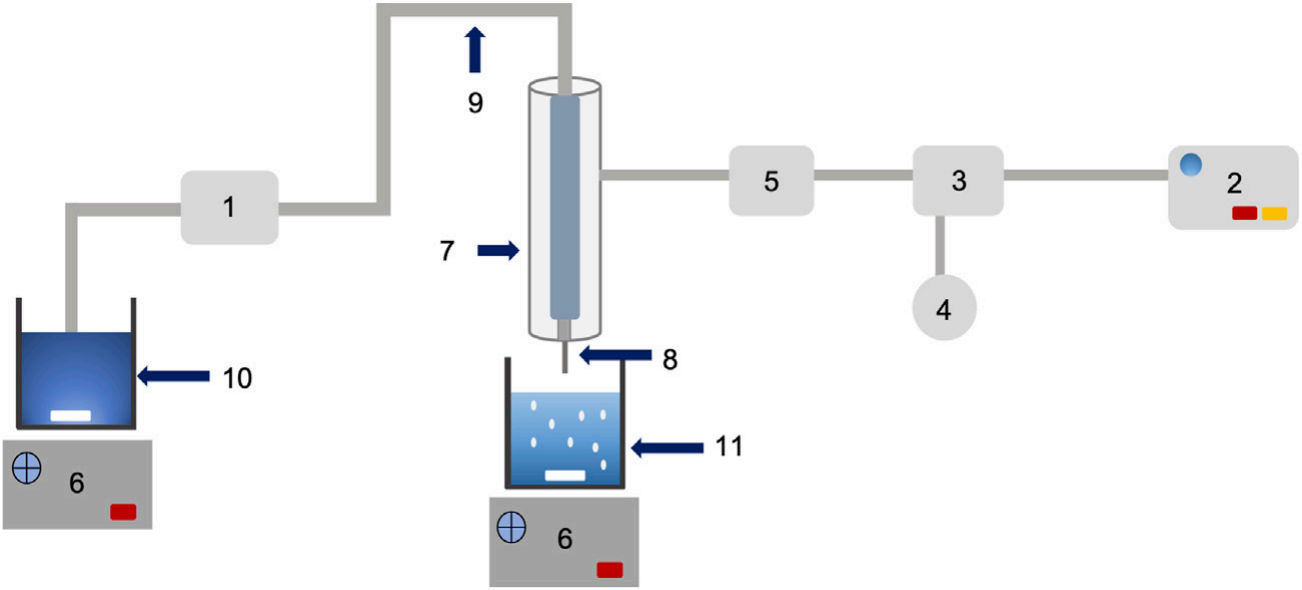
Schematic diagram and working of bead making unit (designed and fabricated of low cost encapsulation machine): peristaltic pump (1); air pump (2); pressure regulator (3); pressure gauge (4); air filter membrane (5); magnetic stirrer control (6); double stainless steel jacket (7); needle (8); rubber tube (9); mixture solution (10); and gelling beaker (11).

The mixed solution (200 ml, Pectin, hi-maize starch, and bromelain) was dropped through an 18 gauge hypodermic needle (WJ309, Shaoxing Wanjia Appliance Co., Ltd., China) at a controlled pump speed of 6 rpm using a peristaltic pump (YZ1515x, Longer Precision Pump Co., Ltd., China) into coagulation bath containing 0.2 M calcium chloride solution (50 ml) using a hydrogel bead making unit (Figure 1), designed and fabricated at the Bioprocess Technology Laboratory workshop, Asian Institute of Technology, Thailand. The hydrogel beads formed were further incubated in the same coagulation fluid for 30 min with stirring (50 rpm). The subsequent hydrogel beads were isolated and washed in excess normal saline solution (0.9% w/v NaCl) before drying at room temperature (25°C) for 24 h. The prepared hydrogel beads were coded as P1, P2, P3, P4, and P5, and the characteristics of hydrogel beads are shown in Table 1. Three batches of each hydrogel beads were produced for further analysis.

### Particle Size Determination of Hydrogel Beads

The particle size was determined with a micrometer (Mittotuyo micrometer, NSK Co. Ltd., Japan) and expressed as the average of 100 hydrogel beads.

### Determination of Encapsulation Efficiency of Bromelain

The bromelain content in the hydrogel beads was analyzed by following the method described by Anal and Stevens (2005), with some modifications. The bromelain-loaded hydrogel beads (0.1 g) were pulverized and incubated in a 0.02 M phosphate buffer (10 ml, pH 7.4) for 24 h at room temperature (25°C) with 100 rpm shaking. The suspension was further centrifuged (CN-2060, Hsiang Tai Co. Ltd., Taiwan) at 4,000 × g for 30 min. The protein content of the supernatant was measured using a UV-vis spectrophotometer (UNICAM, UV/Vis Spectrophotometer, United Kingdom) at 595 nm. The supernatant from the empty hydrogel beads (without bromelain) was used as a blank. The encapsulation efficiency was calculated from the actual bromelain loading of the hydrogel beads and the theoretical bromelain loading using the formula provided in Eq. 1. Three replicates of each experiment were conducted, and the average values were recorded.

$$\mathrm{Encapsulation efficiency}\;\left(\%\right)=\frac{\mathrm{Bromelain loading}\;\left(g\right)}{\text{theoretical loading }\left(g\right)}x\;100.$$ (1)

Theoretical loading corresponds to the initial amount of bromelain used in the pectin/hi-maize hydrogel beads preparation.

### Swelling Ratio of Dried Hydrogel Beads

The swelling ratio of hydrogel beads was determined by following the methods described by Wang et al. (2019), with slight modifications. Three swelling media were chosen for this study: distilled water, simulated gastric (SGF, pH 1.2), and intestinal (SIF, pH 7.4), without adding digestive enzymes. The dried hydrogel beads (0.1 g) were weighed using an analytical balance (Mettler Toledo, United States) and immersed in different swelling media at 25°C. After a fixed duration of intervals (30, 60, 120, and 240 min), the swollen hydrogel beads were carefully separated from the medium, and the water adsorbed on the hydrogel bead surface was wiped away with a filter paper. After sorption, the weight of the hydrogel beads was evaluated, and their swelling percentage was calculated as explained by Eq. 2:

$$S=\frac{\mathrm{Wt}-\mathrm{Wo}}{\mathrm{Wo}}x\;100,$$ (2)

where S is the percentage of swelling of the hydrogel beads, while W_t_ and W_o_ are the weight of swollen hydrogel beads at a fixed time interval and the initial weight of the hydrogel beads, respectively.

### Surface Morphology of Pectin/Hi-Maize Starch Hydrogel Beads

A scanning electron microscope (SEM) (SU5000 EM Wizard, Hitachi, Japan) was used to evaluate the morphology and surface appearance of hydrogel beads. The samples were mounted to a specimen stub with double-sided tape and coated with a thin layer of gold on an ion sputter coater to improve their visibility and sufficient resolution. Additionally, the physical appearance of the hydrogel beads was observed through a digital camera (DSC-W530, Sony Corporation, Japan).

### Fourier Transform Infrared Spectroscopy Analysis

Infrared spectra of the pectin, hi-maize starch, bromelain, and encapsulated bromelain in pectin/hi-maize starch were measured using FTIR spectrophotometer (Nicolet iS50, Thermo Scientific, United States). The pellets were scanned in the range of 4,000–400 cm^−1^, and spectra of all samples were collected and analyzed using the system software (OMNIC software).

### Disintegration of Hydrogel Beads

The disintegration of the hydrogel beads in SIF (pH 7.4) was studied by following the method described by Anal and Stevens (2005), with some modifications. Hydrogel beads (0.1 g) were preincubated with 10 ml of SGF (pH 1.2) for 2 h at 37°C in an incubator (JSGI-150T, JS Research Inc., Korea) with 100 rpm shaking. Following filtering, the swollen hydrogel beads were transferred to another vial containing 10 ml of SIF (pH 7.4). The samples were further incubated at 37°C at 100 rpm in a shaking incubator (JSGI-150T, JS Research Inc., Korea). The time required for complete disintegration was measured. Triplicate examinations were performed on all of the samples.

### *In Vitro* Bromelain Release Studies

*In vitro* release of entrapped bromelain was performed using a conical flask (125 ml) by following the methods described by Zhang et al. (2017) and Anal et al. (2003), with slight modifications. The release media were SGF (pH 1.2) and SIF (pH 7.4) maintained at 37 ± 1°C in an incubator (JSGI-150T, JS Research Inc., Korea) under a constant rotation speed of 100 rpm. Further, the hydrogel beads (0.1 g) were placed into a beaker flask with 20 ml of SGF medium (pH 1.2) for 2 h, and subsequently in the same volume of SIF media (pH 7.4) for 3 h. Additionally, 1 ml of solution was collected from the release medium at regular intervals and immediately replaced by the same volume of fresh medium. After removing debris by centrifugation (CN-2060, Hsiang Tai Co. Ltd., Taiwan) at 4,000 × g for 30 min, bromelain in the release medium was directly evaluated using a UV-vis spectrophotometer (UNICAM, UV/Vis Spectrophotometer, United Kingdom) for measurement of protein content by Bio-Rad Bradford assay at the wavelength of 595 nm using bovine serum albumin (BSA) as a reference standard. The empty pectin/hi-maize hydrogel beads (without bromelain) in pepsin solution were used as a blank in the SGF study, and the empty pectin/hi-maize hydrogel beads (without bromelain) in pancreatin solution was used as a blank in the SIF study.

### Effect of Temperature on the Activities of Non-Encapsulated and Encapsulated Bromelain

The thermal stability test was conducted by following the method described by Naghdi et al. (2019), with some modifications. Free bromelain (2 mg; 0.6 Unit/mL) and 100 mg of encapsulated bromelain in dried hydrogel beads were added to separate tubes containing 2 ml of phosphate buffer saline (PBS) (at constant pH 7.4) and incubated in a water bath (SV 1422, Memmert GmbH + Co. KG, Germany) at 30, 65, 72, and 95°C for 1, 2, 3, 4, and 5 min. Following the incubation period, aliquots were taken at various time intervals, and the samples were cooled to 25°C. The bromelain activity of free and encapsulated samples was measured spectrophotometrically (UNICAM, UV/Vis Spectrophotometer, United Kingdom) at 660 nm based on the method explained in bromelain activity determination. Subsequently, the residual protease activity was measured, and the relative proteolytic activity (%) was further calculated through Eq. 3:

$$Relative\;proteolytic\;activity\;\left(\%\right)=\frac{Enzyme\;activity\;of\;\;treated\;samples\;x\;100}{\;Enzyme\;activity\;of\;\;untreated\;sample}.$$ (3)

### Bromelain Activity Determination

The proteolytic activity of the solution was determined using casein and L-tyrosine as a substrate and a standard, respectively, following the method described by Mala et al. (2021b), with slight modifications. Based on this assay, the casein was hydrolyzed by bromelain, resulting in the release of L-tyrosine. Under standard assay conditions, the amount of enzyme that produced a product equal to 1 µg of tyrosine min^−1^ ml^−1^ was described as one unit of enzyme activity, which can be expressed as Units/ml. The enzyme solution (1 ml) was mixed with casein (5 ml, 0.65% w/v) in 0.05 M potassium phosphate buffer (0.05 M, pH 7.5). The reaction was performed at 37°C in a water bath (SV 1422, Memmert GmbH + Co. KG, Germany) for 10 min. Subsequently, the reaction was terminated by the addition of trichloroacetic acid (5 ml, 0.1 M). After that, the mixture was then filtered through a syringe filter (0.45 µm diameter) (SC13P045S, HyundaiMicro, Korea). The filtrate (2 ml) was mixed with 5 ml of 0.5 M sodium carbonate solution and 1 ml of 20% (v/v) Folin Ciocalteu’s phenol reagent and incubated for 30 min in a water bath at 37°C. Then the solution was filtered through a syringe filter (0.45 µm diameter) (SC13P045S, HyundaiMicro, Korea), and the absorbance of the mixture was measured at 660 nm with a UV-vis spectrophotometer (UNICAM, UV/Vis Spectrophotometer, United Kingdom). The bromelain activity was calculated as Eq. 4:

$$\mathrm{Units}/\mathrm{ml enzyme}\;=\frac{\left(\mathrm{µmole tyrosine equivalents released}\right)\left(11\right)}{\left(1\right)\left(10\right)\left(2\right)},$$ (4)

where 11 = total volume (in milliliters) of assay, 10 = time of assay (in minutes) as per the unit definition, 1 = volume of enzyme (in milliliter) of enzyme used, and 2 = volume (in milliliters) used in colorimetric determination.

### Statistical Analysis

The experiments were performed in three replicates, and the data were presented as mean with standard deviation. A statistical analysis was performed using a commercial statistical package (SPSS Version 22, Chicago, IL, United States) in which ANOVA and Tukey’s HSD test were conducted to evaluate the significant differences (*p* < 0.05) among mean observations.

## Results and Discussion

### Bromelain-Loaded Pectin/Hi-Maize Starch Hydrogel Beads

In this study, bromelain was encapsulated in a pectin/hi-maize starch hydrogel using an extrusion method. Intermolecular cross-linking between Ca^2+^ ions and the negatively charged carboxylate (-COO-) on the pectin backbone was used to create hydrogels. Divalent metals promote polyanion–cation–polyanion interactions between pairs of carboxylate groups on neighboring helices of the pectin structure (Patel et al., 2014; Dafe et al., 2017). Pectin and pectin/hi-maize starch hydrogel beads showed mean particle size values ranging from 3,873 to 4,115 µm (Table 1). The size of the hydrogel beads produced by the extrusion technique varied significantly (*p* < 0.05) in the presence of different pectin and hi-maize starch ratios.

Additionally, previous studies indicated that the size of the hydrogel beads varied depending on the material used (Anal and Stevens, 2005). It can be observed that the incorporation of hi-maize starch filler resulted in reduction in particle size, as illustrated in Figure 3. Dafe et al. (2017) also reported the moderate decrease of particle size with the increase of hi-maize starch content. However, Zafeiri et al. (2021) demonstrated that the quantity of starch encapsulated in the alginate hydrogel beads decreases in size as the amount of starch utilized to produce the formulation rises. However, the starch-rich alginate beads are more resistant to deformation than the starch-free alginate beads because the native starch granules serve as mechanically inactive filler particles.

### Encapsulation Efficiency of Bromelain in Hydrogel Beads

The encapsulation efficiency (Table 1) was determined based on the ratio amount of bromelain loading after encapsulation over the initial amount of bromelain. Bromelain entrapment in pectin/hi-maize starch hydrogel beads was significantly affected by variations in pectin and hi-maize starch concentrations. The encapsulation efficiency improves significantly as the hi-maize starch content increases. The hydrogel beads made of only pectin (P1) had an encapsulation efficiency of 67.66 ± 2.06%. This occurs due to inadequate cross-linking and a large pore size, which allows the entrapped active ingredients to diffuse out during and after gelation (Anal and Stevens, 2005). Moreover, the entrapment of bromelain was also low (71.29%) at the low hi-maize starch concentration of P2 formulation.

The formulation P4 had a maximum encapsulation efficiency of 80.53 ± 1.51%, which can be attributed to the high amount of modified starch interacting with pectin, which generates stability between the two polysaccharides to retain the highest bioactive content in the polymer matrix of the microcapsule formed (Basilio Cortes et al., 2021).

The obtained values showed a high range of encapsulation efficiency values compared to earlier reports for bromelain encapsulation in materials such as chitosan nanoparticles, chitosan nanofibers, and katira gum as carriers (Bernela et al., 2016; Bayat et al., 2019; Ataide et al., 2021). Martin et al. (2013) revealed the increased encapsulation efficiency of *Lactobacillus fermentum CECT5716Lb* Casei cells with increased concentration of starch in alginate–starch–calcium chloride–based hydrogel beads. Similarly, Pankasemsuk et al. (2016) demonstrated that an alginate bead containing 1% (w/w) hi-maize starch provided higher viable cells for both gastric and bile fluids than alginate beads alone. Hosseini et al. (2014) revealed that adding resistant starch to alginate–starch hydrogel beads improved nisin-loading capacity values compared to alginate beads alone.

### Swelling Ratio of Dried Hydrogel Beads

Swelling is regarded as the initial step towards releasing the enclosed material and disintegration of the hydrogel beads. The difference in osmotic pressure between the fluid within and outside the hydrogel beads causes the hydrogel beads to swell (Kumaree et al., 2015). In addition, polysaccharides have a high affinity for water, allowing encapsulated active ingredients to disperse out rapidly. In this case, cross-linking with an inorganic compound is used to minimize the permeability of polysaccharides (Arica et al., 2002). As a polyanion natural polymer, pectin has a strong ability to associate with cations including Ca^2+^ or Sr^2+^, promoting the gel network structure by intermolecular cross-links between the calcium ions (Ca^2+^) and the negatively charged carboxyl groups (COO^−^). The ratio and composition of ionic and non-ionic functional groups in hydrogels determine their swelling potential (Mahdavinia et al., 2015; Dafe et al., 2017).

**FIGURE 2:**
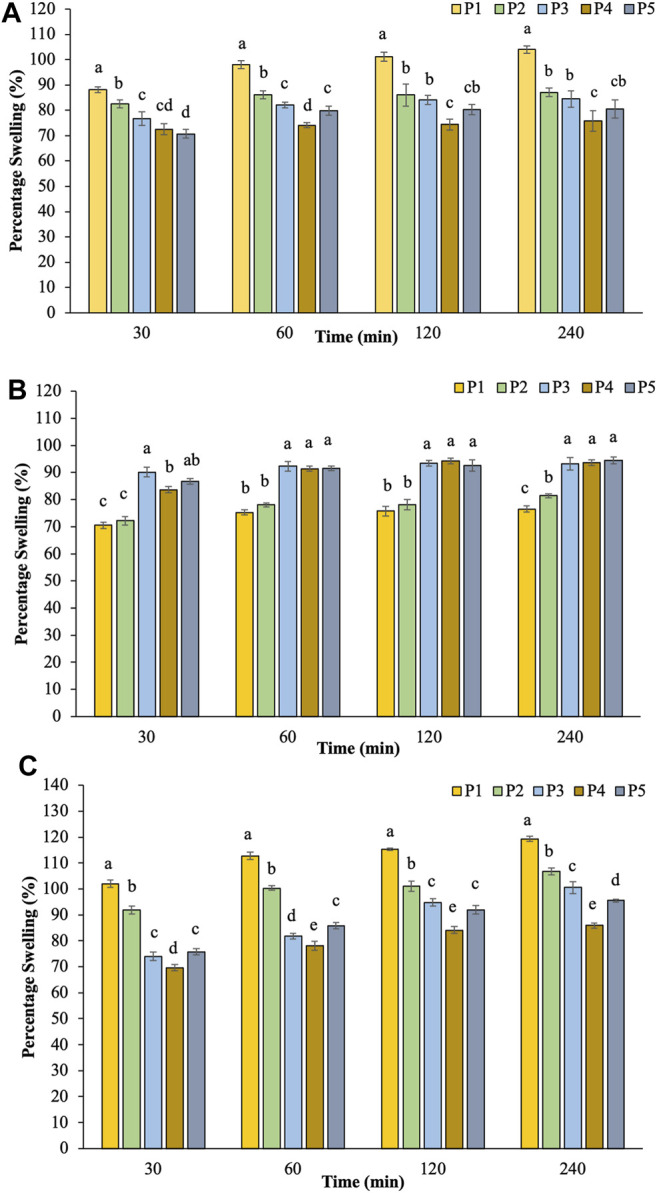
The percentage swelling of hydrogel beads in water **(A)**, pH 1.2 **(B)**, and pH 7.4 **(C)**.

The dynamic swelling behavior of hydrogel beads in distilled water is illustrated in Figure 2A. The rate of water absorption increased dramatically at the first 30 min and then started to level off. The swelling degree of hydrogels indicated a systemic pattern attributed to their compositions. The percentage swelling in water decreased with increasing the ratio of hi-maize starch content.

The swelling capacity of prepared hydrogel beads was further investigated at pH 1.2 and pH 7.4 to simulate the gastrointestinal conditions (Dafe et al., 2017; Zhu et al., 2019). Figure 2B illustrates the results of percentage swelling in simulated gastric juice pH 1.2. The swelling capability gradually increased initially for 30 min and then reached the saturation level without any evidence of disintegration during the incubation time. The findings revealed that the swelling index of pectin/hi-maize starch hydrogel beads (P3–P5) increased up to 93.30–94.40% during 4 h. On the other hand, pectin hydrogel beads (P1) exhibited a lower swelling rate of 76.58%. Maximum swelling capacities were obtained for pectin/hi-maize starch hydrogel beads than pectin hydrogel beads alone. Since the carboxyl groups of pectin are protonated in an acidic medium, the hydrogen bonding and hydrophobic forces between or within pectin molecules result in a more compact structure and lower swelling capacity (Chang and Lin, 2000; Dafe et al., 2017).

In the following sequence of tests, swollen hydrogel beads in the gastric fluid were subsequently incubated in PBS (pH 7.4), as shown in Figure 2C. Hydrogel beads prepared without hi-maize starch in P1 presented a swelling index up to 119.33%. The addition of hi-maize starch (P3–P5) resulted in stronger hydrogel beads with decreased swelling index to 85.92–106.82%. The swelling pattern of pectin/hi-maize starch hydrogel beads is significantly influenced (*p* < 0.05) by pectin and hi-maize starch concentrations. A combination of 1.5% hi-maize starch (P4) played an important role in percentage swelling at 85.92%. Carboxylic acid groups on pectin chains convert to negatively charged carboxylate ions in basic and neutral environments. Consequently, electrostatic repulsion between the deprotonated carboxyl groups causes the network to expand and swell (Dafe et al., 2017). The combination of 1.5% hi-maize starch (P4) provided stronger protection than the lower and higher concentrations. This is related to the protective effect of hi-maize starch at appropriate concentrations against media penetration (Pankasemsuk et al., 2016).

### Surface Morphology of Pectin/Hi-Maize Starch Hydrogel Beads

**FIGURE 3:**
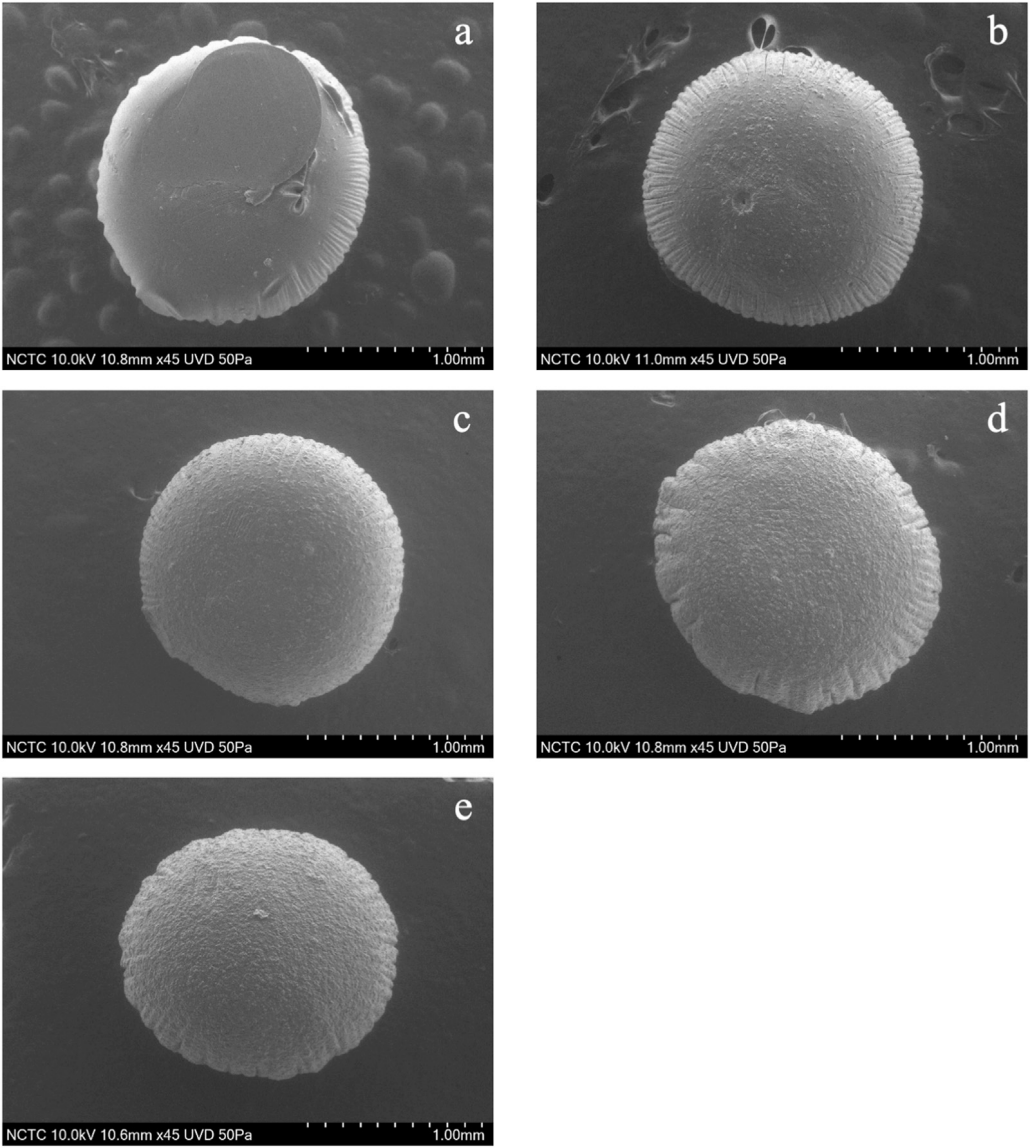
Scanning electron microscopy (SEM) images (magnification ×50) showing the surface morphology of pectin/hi-maize starch hydrogel beads with different pectin:hi-maize starch ratios: **(A)** bead P1 (6: 0), **(B)** bead P2 (5.5: 0.5), **(C)** bead P3 (5: 1), **(D)** bead P4 (4.5: 1.5), and **(E)** bead P5 (4: 2).

**FIGURE 4:**
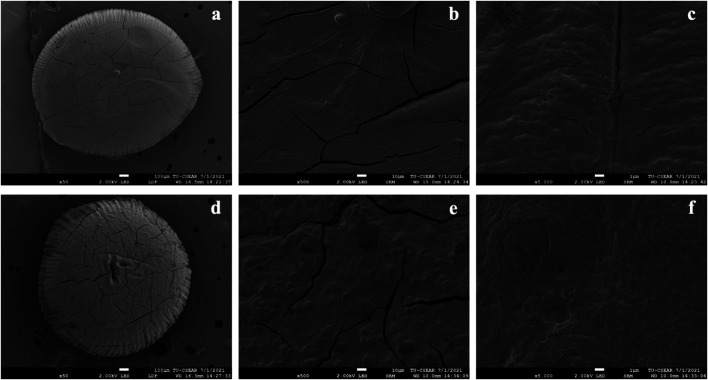
Scanning electron microscopy (SEM) images showing the surface morphology of pectin/hi-maize starch hydrogel beads. **(A)** Only pectin hydrogel bead (magnification ×50), **(B)** only pectin hydrogel bead (magnification ×500), **(C)** only pectin hydrogel bead (magnification ×5000), **(D)** pectin/hi-maize starch hydrogel bead (ratio 4.5:1.5; magnification ×50), **(E)** pectin/hi-maize starch hydrogel bead (ratio 4.5:1.5; magnification ×500), and **(F)** pectin/hi-maize starch hydrogel bead (ratio 4.5:1.5; magnification ×5000).

The shape and sizes of pectin hydrogel beads (Figure 3A) show significant differences from pectin/hi-maize starch hydrogel beads (Figures 3B–E). The addition of hi-maize starch in the pectin matrix resulted in a more packed and spherical shape, as evidenced by the surface morphology of the hydrogel beads (Figures 3, 4). This is mainly due to homogeneously dispersed starch, which can maintain interstitial spaces in the matrix, promoting the pectin/hi-maize starch hydrogel beads structure and preventing cracking and shrinkage (Córdoba et al., 2014). Hosseini et al. (2014) reported a similar observation, indicating that starch serves as structural support, thus limiting shrinkage and preserving the spherical form of dried hydrogel beads. This indicates that hi-maize starch encouraged a more adhesive reaction than the hydrogel bead without starch. In addition, Pankasemsuk et al. (2016) demonstrated that alginate along with 1% hi-maize starch matrix appeared more compact with probiotic cells. The application of hi-maize starch and alginate for coating bacterial cells allowed for a prebiotic ability from the starch and the adhesion of more probiotic cells into the hydrogel beads (Anal and Singh, 2007).

**FIGURE 5:**
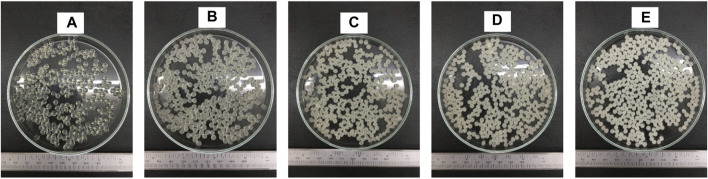
Digital photo of pectin/hi-maize starch hydrogel beads with different pectin:hi-maize starch ratios: **(A)** bead P1 (6: 0), **(B)** bead P2 (5.5: 0.5), **(C)** bead P3 (5: 1), **(D)** bead P4 (4.5: 1.5), and **(E)** bead P5 (4: 2).

Figure 5 illustrates the shape and appearance of the hydrogel beads. The pectin hydrogel beads without starch contents were clear and transparent. The cracks were found on the surface of hydrogel beads when the hi-maize starch was applied along with pectin. This was related to hi-maize starch’s insolubility in pectin solution. Furthermore, when hi-maize starch was applied, the pectin/hi-maize hydrogel beads showed a uniform distribution of hi-maize starch and provided an opaque appearance.

### Fourier-Transforms Infrared Spectroscopic Studies

**FIGURE 6:**
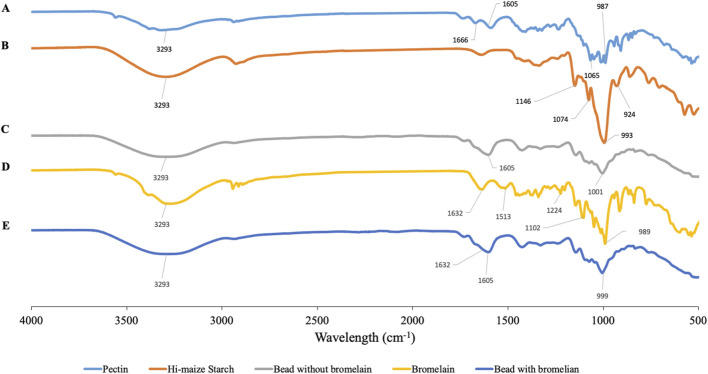
FTIR spectra of pectin **(A)**, hi-maize starch **(B)**, hydrogel bead without bromelain **(C)**, bromelain **(D)**, and bromelain-loaded hydrogel bead **(E)**.

The FTIR spectra of pectin, hi-maize starch, bromelain, and hydrogel beads with bromelain and without bromelain loading are presented in Figure 6. Pectin shows characteristic spectra corresponding to the polysaccharide chemical structures. The intense bands at 3,600–3,000 cm^−1^ are attributed to the characteristic band for the stretching vibration of the -OH group. The bands at 1700–1,600 and 1,210–1,050 cm^−1^ are attributed to the intramolecular hydrogen bond and C-O stretching, respectively. The vibrational absorption peaks of the C-H bond of monosaccharide are observed at 1,000–850 cm^−1^ (Zhu et al., 2019).

In hi-maize starch, the broad peak at a wavelength around 3,300–3,200 cm^−1^ represents -OH stretching vibrations (Ahmad et al., 2016), and regions between 995–985 cm^−1^ are usually associated with stretching or bending vibrations of C=C. The intense bands at 1,085–1,050 cm^−1^ are attributed to the characteristic band for the stretching vibration of the C-O stretching group (Yu and Huang, 2010; Ahmad et al., 2019).

The spectra of bromelain show a peak at 3,400–3,300 cm^−1^ showing the existence of NH–stretching vibrations. The peak at 1,632 cm^−1^ shows the presence of C=O stretching groups (amide I region at 1,690–1,600 cm^−1^). The characteristic of C-N stretching vibration frequencies are assigned to IR peaks at 1,513, 1,224, and 1,102 cm^−1^. Bernela et al. (2016) reported the appearance of a similar band of bromelain encapsulated in katira gum nanoparticles.

Bromelain-loaded hydrogel beads exhibited the characteristic peaks of bromelain, pectin, and hi-maize starch. Similar peaks with a broad range were observed in the bromelain hydrogel bead, which represents the encapsulation of bromelain in the pectin–starch matrix

without any evident interaction (Bernela et al., 2016). The slight shift of these wavenumber frequencies was observed in bromelain-loaded hydrogel beads. The hydrogel bead composited with bromelain is determined by a band at 1,632 cm^−1^ (COO-Ca) corresponding to pectin (Khorasani and Shojaosadati, 2017). Previous studies have reported that hydrogen bonding is considered as the major interaction between phenolic compounds and starch-based wall materials (Liu et al., 2011; Ahmad et al., 2019).

### Disintegration of Hydrogel Beads

**FIGURE 7:**
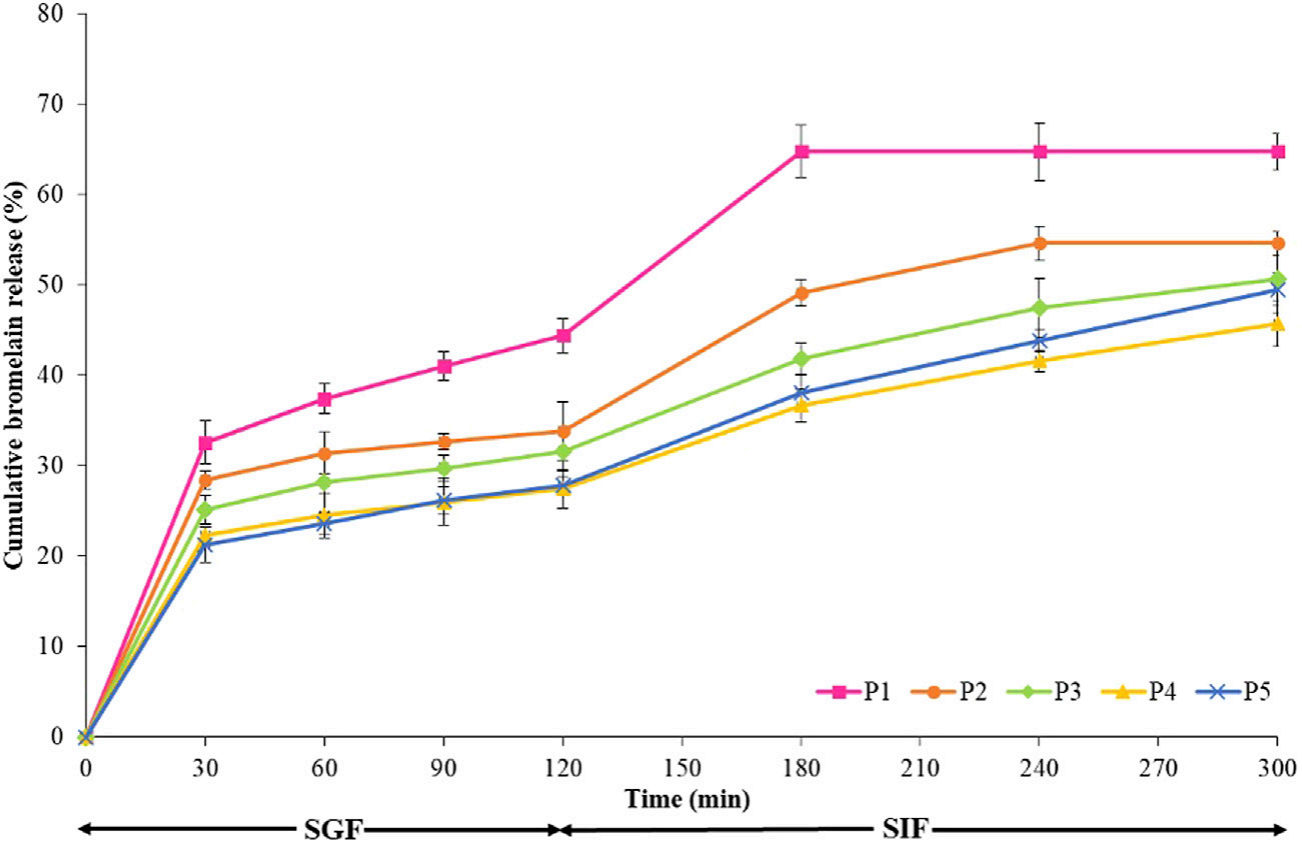
Bromelain release behavior of the hydrogel beads in the simulated gastric fluid (SGF) for 2 h and in simulated intestinal fluid (SIF) for 3 h at 37°C with a stir rate of 100 rpm.

The disintegration of hydrogel beads was investigated in SIF (pH 7.4). The term “hydrogel bead disintegration” refers to the loss of the bead-like structure as observed under a microscope (Anal et al., 2006). The time needed for complete disintegration of various hydrogel beads, prepared by the extrusion method, is presented in Table 1. Each sample exhibited a different profile of dissolving in the dissolution media. The hydrogel beads prepared in formulation P4 showed a delay in disintegration and consequently a slow release of bromelain (Figure 7). The pectin hydrogel beads (P1) swollen in SGF appeared not to be stable for long periods in the intestinal fluid. In SIF, the pectin hydrogel beads completely disintegrated by bursting within an hour. In all types of hydrogel beads, the presence of hi-maize starch in the pectin/hi-maize starch hydrogel beads delayed the disintegration and retarded the erosion process. However, the presence of insufficient resistant starch resulted in a lack of stability and, therefore, a reduced protective impact. However, an excessive amount of resistant starch may have been produced as a result of a thick starch layer causing weakness in the coating material (Pankasemsuk et al., 2016); as a result, a combination of 2% hi-maize starch hydrogel bead (P5) was found to be less stable over longer periods of time than a combination of 1.5% hi-maize starch hydrogel bead (P4). Disintegration time is also influenced by the pH of the medium (Anal et al., 2006). After their transfer to neutral pH in SIF, the hydroxyl ions tend to displace the anionic pectin in the calcium–pectin complex, and as a consequence, the complex dissociates and the matrix erodes (Bhopatkar et al., 2005; Dafe et al., 2017).

### *In Vitro* Bromelain Release Studies

*In vitro* release experiments were conducted to identify variations in the release profiles of hydrogel beads. The release of the bromelain from the pectin/hi-maize starch is strongly affected by the pH of the medium. Bromelain is a water-soluble polymer that readily diffuses out (Chakraborty et al., 2021).

Figure 7 illustrates the release profile of encapsulated bromelain in both SGF and SIF simulated conditions for pectin and various pectin/hi-maize starch matrix formulations. The pectin and pectin/hi-maize hydrogel beads were incubated in SGF (pH 1.2) for 2 h and then in SIF (pH 7.4) for the next 3 h. Bromelain release from pectin hydrogel beads (P1) was around 44.37% in SGF after 2 h (Figure 7). Pectin hydrogel beads that were not strengthened with hi-maize starch most likely lacked the necessary cross-linking density to prevent entrapment from diffusing out. Conversely, during the first 2 h in SGF, the pectin/hi-maize starch (P2–P5) significantly decreased the release of entrapped bromelain and the release of entrapped bromelain hydrogel beads were reduced to 27.41–33.85%. First, there is a rapid release step, in which bromelain is physically entrapped on the surface of hydrogel beads, which was covered by a very thin coating of materials. Furthermore, the rapid initial release is due to the potential uptake of water into the hydrogel beads, as well as the volume expansion of the matrix as a result of the water uptake. This second stage is caused by polymer degradation or bromelain diffusion from the inside to the outside (Hosseini et al., 2014; Zhu et al., 2019).

The hydrogel beads were further transferred to SIF after 2 h in SGF. Within an hour in SIF, the pectin hydrogel beads (P1) disintegrated and lost all remaining bromelain. A statistical analysis of the release after 3 h in SIF reveals that increasing the content of hi-maize starch considerably decreases the release of bromelain. As shown in Figure 7, the release behavior of pectin hydrogel beads (P1), up to 64.69% of bromelain contained in the hydrogel beads, was released after passage through the gastrointestinal system. The pectin/hi-maize starch hydrogel beads in P4 showed only a 45.65% release within 3 h in SIF; this release pattern is suited for controlled release. Furthermore, pectin/hi-maize starch in formulations P3–P5 remained stable for around 2–3 h, whereas pectin/hi-maize hydrogel beads P4 released bromelain continuously for more than 3 h. The delay in releasing hydrogel beads containing hi-maize starch correlates with the delay in the erosion and disintegration of hydrogel beads.

The addition of hi-maize starch to the hydrogel beads prolonged the period of release. The slower erosion and consequently more prolonged release of bromelain from pectin/hi-maize starch hydrogel beads most certainly affect the beads strengthening by starch. The hydrogel network becomes denser as the concentration of starch in the hydrogel matrix increases, which may decrease the rate of solution diffusion through the hydrogel.

Bromelain encapsulated in hydrogels released at a faster rate at pH 7.4 (SIF) than at pH 1.2 (SGF). The carboxylic acid group on the backbone of pectin transforms into negatively charged carboxylate ions at higher pH values (pH 7.4); as a result of electrostatic repulsion between carboxylate ions, the network expands and bromelain diffusion from the pectin/starch hydrogels increases. Under acidic conditions, the anionic carboxylate (pKa∼4.5) on the pectin backbone is transformed to neutral carboxylic acid, resulting in a hydrogen-bonded network (Dafe et al., 2017). Similarly, the greater release from Ca–alginate beads is attributed to the presence of phosphate ions in SIF, which chelates the Ca^2+^ at neutral pH (Anal et al., 2003; Bhopatkar et al., 2005). Previous research indicated that the release of macromolecules such as bovine serum albumin is mostly caused by hydrogel bead erosion. Erosion is most possibly influenced by a reduction in the number of cross-links and the dissolution of the ionotropic coherence in the hydrogel beads (Anal et al., 2003; Bhopatkar et al., 2005).

The presence of resistant starch in the alginate matrix slowed the release of nisin, resulting in a higher time required value for alginate/hi-maize microparticles (Hosseini et al., 2014). In addition, a mixture of 1% hi-maize starch resulted in optimum cell survivability (Pankasemsuk et al., 2016).

### Effect of Temperature on the Activities of Non-Encapsulated and Encapsulated Bromelain

**FIGURE 8:**
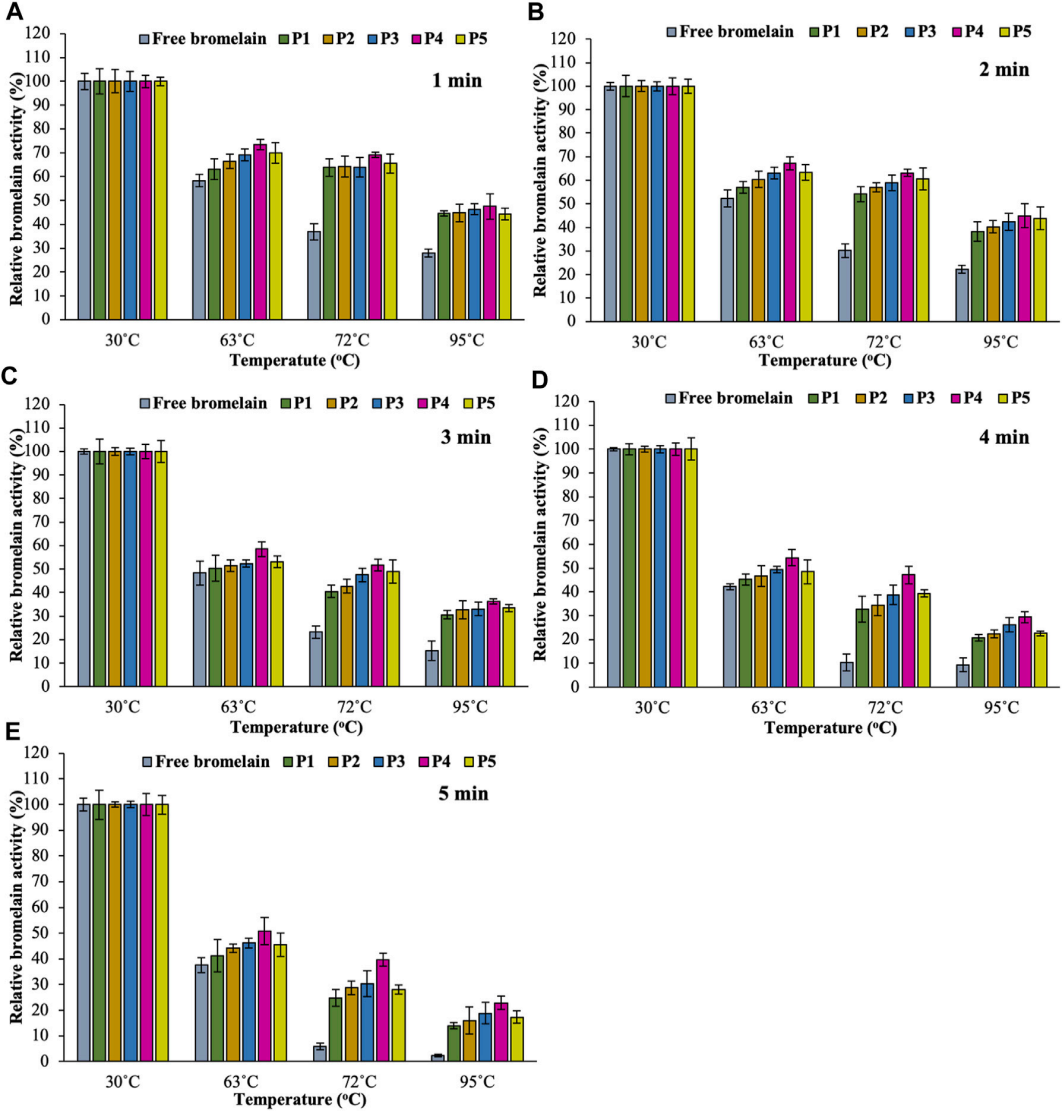
Effect of temperature (in PBS at pH 7.4) on the activity of free bromelain and encapsulated bromelain (P1, P2, P3, P4, and P5) for 1 min **(A)**, 2 min **(B)**, 3 min **(C)**, 4 min **(D),** and 5 min **(E)** in an incubator with 100 rpm shaking.

Encapsulation of enzymes in hydrogel beads provides some protection against temperature variations, which results in increased stability as compared to free enzyme. Improving this property allows the systems for manufacturing applications in the operating temperature ranges (Gassara-Chatti et al., 2013; Naghdi et al., 2019). An Enzyme with a matrix generally increases enzyme’s thermostability resulting from interactions between the enzyme and the support, which enhances molecular rigidity (Kunamneni et al., 2008; Ko et al., 2012). Thermostability of free and encapsulated enzyme was evaluated by incubating free bromelain and encapsulated bromelain in various hydrogel beads at 30, 63, 72, and 95°C for 1, 2, 3, 4, and 5 min, and the results are summarized in Figure 8. After heat treatment, both free and all hydrogel beads lost activity, especially at higher temperatures. However, the thermostability of the encapsulated bromelain hydrogel beads was better than the free enzyme.

While considering the temperature range of 30–95°C, bromelain activity was found highest at 30°C for all hydrogel bead formulations. All the free bromelain showed the lowest relative activity (%) at all temperatures. On the other hand, encapsulated bromelain significantly increased bromelain activity in pectin/hi-maize starch hydrogel beads. Increasing the content of hi-maize starch at a different ratio of pectin content (i.e., pectin/hi maize starch ratios from 6:0 to 4:2% w/w) also enhanced the bromelain activity. However, the encapsulated bromelain in the pectin/hi-maize starch hydrogel beads produced at a pectin/hi-maize ratio of 4.5:1.5 percent w/w (formulation P4) obtained the highest relative bromelain activity in all heat treatments. The pectin and hi-maize starch hydrogel beads at optimal concentration have reduced porosity and a thicker structure, which results in the hydrogel bead limiting solution entry (Koo et al., 2001). Furthermore, the starch-coated hydrogel provided superior protection for the encapsulated active compounds. Specifically, this feature results from a tightly packed environment given by hydrogels, which effectively shields the active compounds from external stress conditions such as oxygen, heat, and humidity (Dafe et al., 2017). The alginate–soy protein isolate hydrogel beads containing *L. plantarum* cells in mango juice demonstrated effective resistance against thermal treatment (72°C for 90 s). Moreover, probiotic bacteria encapsulated in chitosan–alginate beads and alginate–fish gelatin protein were reported to be more resistant to stress conditions associated with food processing and high temperatures related to pasteurization, while retaining their functional characteristics (De Prisco et al., 2015; Kumaree et al., 2015).

## Conclusion

Encapsulated beads of bromelain were successfully obtained by the extrusion gelation method of various ratios of two different biopolymers, pectin and resistant starch with different ratios into the gelation solution. The encapsulated bromelain in hydrogel beads developed with the combination of these polyelectrolytes can protect bromelain from harsh gastric conditions (lower pH) and higher processing temperatures. The presence of hi-maize starch at a concentration of 1.5% w/w in pectin 4.5% w/w formulation resulted in a higher percentage of encapsulation efficiency compared to the formula of pectin alone. The application of resistant starch blending had a significant influence on the extended and gradual release as well as activity of bromelain. Furthermore, the combination of resistant starch with pectin biopolymer significantly protected bromelain from the extreme environments of high temperature processing. Due to high efficiency in protection and controlled release carrier for bromelain, bromelain-loaded pectin and resistant starch hydrogel beads can be applied for bromelain delivery applications in food and pharmaceutical products development for controlled intestinal release.

## Data Availability

The original contributions presented in the study are included in the article/Supplementary Material; further inquiries can be directed to the corresponding author.
